# Incremental Learning to Personalize Human Activity Recognition Models: The Importance of Human AI Collaboration †

**DOI:** 10.3390/s19235151

**Published:** 2019-11-25

**Authors:** Pekka Siirtola, Juha Röning

**Affiliations:** Biomimetics and Intelligent Systems Group, University of Oulu, P.O. BOX 4500, FI-90014 Oulu, Finland; juha.roning@oulu.fi

**Keywords:** personalization, human activity recognition, incremental learning, human AI collaboration

## Abstract

This study presents incremental learning based methods to personalize human activity recognition models. Initially, a user-independent model is used in the recognition process. When a new user starts to use the human activity recognition application, personal streaming data can be gathered. Of course, this data does not have labels. However, there are three different ways to obtain this data: non-supervised, semi-supervised, and supervised. The non-supervised approach relies purely on predicted labels, the supervised approach uses only human intelligence to label the data, and the proposed method for semi-supervised learning is a combination of these two: It uses artificial intelligence (AI) in most cases to label the data but in uncertain cases it relies on human intelligence. After labels are obtained, the personalization process continues by using the streaming data and these labels to update the incremental learning based model, which in this case is Learn++. Learn++ is an ensemble method that can use any classifier as a base classifier, and this study compares three base classifiers: linear discriminant analysis (LDA), quadratic discriminant analysis (QDA), and classification and regression tree (CART). Moreover, three datasets are used in the experiment to show how well the presented method generalizes on different datasets. The results show that personalized models are much more accurate than user-independent models. On average, the recognition rates are: 87.0% using the user-independent model, 89.1% using the non-supervised personalization approach, 94.0% using the semi-supervised personalization approach, and 96.5% using the supervised personalization approach. This means that by relying on predicted labels with high confidence, and asking the user to label only uncertain observations (6.6% of the observations when using LDA, 7.7% when using QDA, and 18.3% using CART), almost as low error rates can be achieved as by using the supervised approach, in which labeling is fully based on human intelligence.

## 1. Introduction

This study focuses on human activity recognition based on inertial sensor data, which can be collected for instance using smartphone sensors. Traditionally, activities are recognized by training a user-independent recognition model relying on data that are given prior to training. The problem of this approach is that it assumes that structure of the data remains static in the future. However, this is not normally the case in real world problems as the world around us constantly changes. On the other hand, in human activity recognition the biggest problem is not the changing world, instead the main problem is the differences between humans: People are unique for instance in terms of physical characteristics, health state or gender. Due to this, studies have shown that a user-independent model that provides accurate results for one person, does not necessarily work as accurately with somebody else’s data. For instance, it was shown in [1] that user-independent models are not accurate if they are trained with healthy study subjects and tested with subjects who have difficulties moving. Therefore, instead of a user-independent model, it would be better to use personal recognition models as they provide more accurate recognition [2,3]. However, the challenge of personal recognition models is that they normally require personal training data, and therefore, a personal data gathering session [4], making this approach unusable out-of-the-box. In addition, there are differences within-users. For instance, gait changes when users get older. Due to this, models should not only be personal, they should also be able to adapt to the changes in a person’s style of performing activities and their environment.

Instead of personal models, also personalized models, which are a combination of user-independent and personal models, can be used in the recognition process. Some personalization methods rely on transfer learning and they require only a small amount of training data from the target user, for instance [5]. Therefore, in these cases, the personal data gathering session can be quite short. However, there are also personalization methods that do not necessarily require a separate personal training data gathering session at all. For instance, Siirtola et al. [6] presented a two step approach to train personalized models. This was especially designed for devices that include several different types of sensors. In the first step, a user-independent model that uses data from all sensors was trained and implemented on the device. When a new person starts to use this device, the implemented user-independent model is used to label the personal streaming data. These predicted labels and gathered streaming data are then used to train a light, energy efficient personal model that uses only a sub-set of the available sensors. This approach improves the recognition accuracy but the problem of the approach is that personalization is based on model retraining. Therefore, all the streaming data and labels needs to be stored to the device’s memory to be able to use them for model training. This obviously is problematic as it requires a good deal of memory to store the data and a large amount of calculation capacity to retrain the model. There are also other studies, where recognition models are personalized in order to improve the model accuracy. Similarly, in [7], model personalization was used to improve the model accuracy. In this case, the personalization was based on the transfer learning algorithm. The study also shows the power of personalization: It was shown that the recognition rates of personalized models are much better than the one’s obtained using non-personalized models.

Incremental learning refers to recognition methods that can learn from streaming data and adapt to new and changing environments. In the case of human activity recognition, this adaptation would mean leaning the personal moving style of a new unseen person, and also adapting to changes in a person’s moving over time, which has been a limitation in many studies, such as [8]. Another advantage of incremental learning is that this adaptation relies on model updating [9]. Therefore, to adapt the model to a new environment and to new users, model retraining is not needed, which has been the requirement in many other activity recognition articles studying model personalization [10]. In fact, incremental learning has already been used in human activity recognition studies [11,12,13]. However, in these studies the focus was not on personalizing the recognition models, however, they do show that inertial sensor-based models benefit from incremental learning as the updated models are more accurate than the original ones. Moreover, [14] used incremental learning-based methods to train models fully relying on personal data. Also in this case it was noticed that updating the model incrementally improves the recognition rate significantly.

For the first time, incremental learning was used to personalize activity recognition models in [15]. In the article it was shown that personalization is possible without a separate data gathering session and without model retraining. Instead, models were continuously updated based on streaming data, and this way, models were adapted to the user’s personal moving style. Moreover, in the article, all of this was done without any user-interruption. This was possible due to using predicted labels, instead of correct ones, in the model updating process. The idea of learning without user-interruption is great from the user experience point-of-view, however, it can also be highly dangerous, and due to concept drift. This type of autonomous learning can also lead to an unwanted end-result. One of the most popular examples of artificial intelligence (AI) learning the wrong things is Microsoft’s Twitter chatbot Tay. The idea of Tay was to mimic a 19-year-old American girl and learn from interactions. Soon however, due to the uncontrolled autonomous self-learning, Twitter users taught to Tay how to be a racist and act like one [16]. This shows the importance of moderating correct learning. It is likely that Tay would not have behaved in such an unwanted way if the developers of Tay had selected which interactions to be used to update Tay’s models and which interactions to ignore.

In [17] incremental learning and active learning were combined for the first time. Unlike in our study, the classification was based on clustering. Moreover, the purpose was more to adapt models to evolving data streams than to personalize models. Therefore, the study does not concentrate on personalizing user-independent models, instead, it adapts one person’s model to other person’s moving style. However, Mannini et al. [18] used an incremental learning based approach to personalize a user-independent human activity recognition model. In the paper, self-learning was not used. Instead, active learning was used to avoid drastic concept drift. This means that only user labeled instances were used in the model updating process. Moreover, users did not have to label all the incoming streaming data. Instead, the uncertainties of the predictions, were measured and the user was asked to label only uncertain observations. These were then used in the updating process. The method did improve the overall accuracy but only a little. The recognition accuracy without personalization was 88.6% and with personalization 89.6%.

This article is an extension to our conference paper [19] where the basic concept for human AI collaboration was presented. This article extends this conference paper in many ways: It gives more insights to the methods, studies in more detail on how to select parameters for the semi-supervised approach, and most importantly, more extensive experiments are made to give a better idea of how well the presented method generalizes on different datasets.

The article is organized as follows: Section 2 introduces the datasets we used and Section 3 explains the process to personalize recognition models. Section 4 concentrates on studying how to select appropriate parameters for the process and Section 5 explains the experimental setup. Section 6 contains the results and discussion, and finally our conclusions are in Section 7.

## 2. Experimental Datasets

The experiments of this study are based on three publicly open data sets. Shoaib et al. [20], which contains data from seven physical activities (walking, sitting, standing, jogging, biking, walking upstairs, and downstairs), and equal amounts of data from each activity. Siirtola et al. [21], which contains data from five physical activities (walking, running, biking, idling, and driving a car). This dataset is imbalanced, 39% of the data is from idling, 20% walking, 9% running, 19% biking, and the rest is from driving a car. Anguita et al. [22], containing data from six activities, standing (19% of the data), sitting (17%), laying down (18%), walking (16%), walking downstairs (14%), and upstairs (16%).

Shoaib data were collected using a smartphone and from five body locations but in this study three body positions are used: arm, waist, and wrist. The data were collected from a 3D accelerometer, 3D gyroscope, and 3D magnetometer using sampling rate of 50 Hz. This study uses data from an accelerometer and gyroscope. The dataset contains measurements from ten study subjects. However, apparently one of the study subjects had placed sensors in a different orientation than the others, making the data totally incompatible to the other subjects’ data. Thus, this person’s data were not used in the experiments, and the final dataset contains nine study subjects. Siirtola data were collected using a Nokia N8 smartphone but only from one body position: trouser pocket. The dataset contains 3D accelerometer data collected from eight study subjects using a sampling rate of 40 Hz. Anguita data was collected using a Samsung Galaxy S2 smartphone with a sampling frequency of 50 Hz. The phone was positioned at the right side of the belt and 3D accelerometer and 3D gyroscope data were gathered. This dataset is much bigger than the other two in the terms of number of study subjects, it contains data from 30 study subjects. However, it is smaller than other two when it comes to the amount of data per user per activity.

A window size of 4.2 s with a 1.4 second slide was used with the Shoaib and Siirtola datasets. However, as Anguita contains much less data per user per activity, in this case a shorter window was used. In fact, with Anguita data a 1.0 s window size with a 1/3 s slide was used. Altogether, Shoaib data consisted of 8980 windows of data, Anguita, 10,689 windows of data, and Siirtola, 7496 windows of data.

From these windows, features were extracted. This study uses features that are commonly used in activity recognition studies including standard deviation, minimum, maximum, median, and different percentiles (10, 25, 75, and 90). Moreover, the sum of values above or below percentile (10, 25, 75, and 90), square sum of values above or below percentile (10, 25, 75, and 90), and number of crossings above or below percentile (10, 25, 75, and 90) were extracted and used as features. In addition, features from frequency domain, for instance sums of small sequences of Fourier-transformed signals, were extracted. In the case of Shoaib data, the features were extracted from raw accelerometer and gyroscope signals, magnitude signals and signals where two out of three accelerometer and gyroscope signals were combined. Anguita and Siirtola datasets were downloaded using OpenHAR [23], which is a Matlab toolbox providing an easy access to accelerometer data of ten publicly open human activity recognition datasets. Therefore, these datasets contained only accelerometer data, and thus, the features were in this case extracted from the raw accelerometer signals, magnitude accelerometer signal, and signals where two out of three accelerometer signals were combined.

## 3. Personalizing Recognition Using Incremental Learning

In this article, an incremental learning based method to personalize human activity recognition models is presented. Ensentially, we studied how much the model updating process benefits from user inputs and when artificial intelligence needs to be supported by human intelligence.

### 3.1. Learn++

For incremental learning, in this article, the Learn++ algorithm [24] is used. It is an ensemble method and the idea of Learn++ is to process incoming streaming data as chunks. For each chunk a new set of weak *base models* are trained and added to a set of previously trained base models through weighted majority voting as an ensemble model [25]. Therefore, the number of base models continuously increases when more streaming data are obtained and new base models are trained based on it.

Learn++ is not the only algorithm that can be used for incremental learning. However, it was chosen to be used in this article as, in [25], it is shown that it is less complex than many other algorithms but still one of the most accurate. This means that Learn++-based classification models can be implemented into devices that do not have much memory and calculation capacity such as wearable sensors. Another advantage of Learn++ is that it can use any classifier as the base classifier. Thus, for this study, it was possible to select base classifiers that are used also in the previous human activity recognition studies. For this study, three base classifiers (linear discriminant analysis (LDA), quadratic discriminant analysis (QDA), and classification and regression tree (CART)) were selected for comparison. Also, these classifiers are very light, therefore, they are also highly suitable for devices with low memory and calculation capacity.

### 3.2. From User-Independent to Personalized Recognition Models

**Figure 1:**
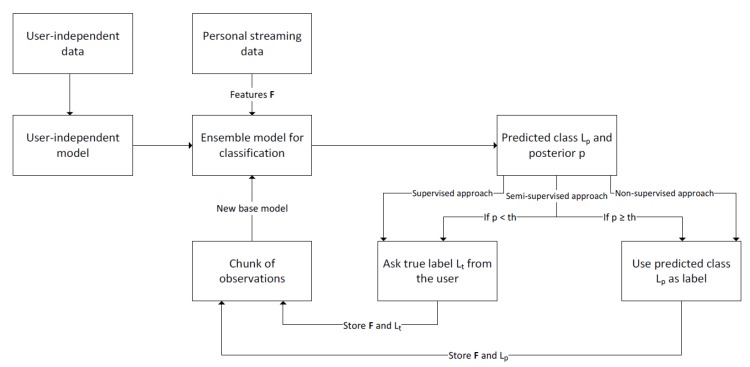
Three approaches to personalize recognition model are compared. The semi-supervised approach is novel and proposed in this study.

The process to personalize a recognition model is presented in Figure 1. Phase 1 of the process is to gather user-independent data to train one or multiple user-independent base models for human activity recognition. These are the first models that are added to the Learn++ model. When this user-independent model is implemented for instance to a wearable device, and subject *X* starts to use the device, personal streaming data from subject *x* can be obtained.

Phase 2 is to start personalizing the Learn++ ensemble model by extracting features from the first chunk of subject x’s streaming dataset. Of course, this data does not have labels. There are three different ways to obtain labels for this data: non-supervised, semi-supervised, and supervised. The non-supervised approach relies on the results of artificial intelligence and only predicted labels were used to update the model [15]. The semi-supervised approach uses both predicted labels and true labels for updating the model, and human intelligence is used to complement artificial intelligence when posterior values of the predicted are below some pre-defined threshold th. Moreover, the supervised approach relies solely on human intelligence, and therefore, each observation is labeled by the user, thus, only true labels are used in the model updating process. Due to this, the supervised approach provides the highest possible accuracy and by comparing these results to the results of other approaches, it can be concluded how optimal the results obtained by other methods are. These three methods are compared in this article.

In phase 2 of the personalization process, after obtaining a chunk of data and labels, features are extracted from the data and one or multiple personal base models are trained based on these. Personal base models are then added to the ensemble. As mentioned, in the classification process Learn++ uses weighted majority voting to combine the results of the base models. These weights are defined for each base model based on the accuracy estimation of the base model, and the accuracy is measured based on the training data and its labels. However, normally incremental learning is used for supervised learning where true labels are available, and thus, the accuracy estimation for base models is easy. This is not the case in this study, as in this study true labels are not available in semi-supervised and non-supervised cases. This means that it is not possible to estimate the accuracy of the new base model. Therefore, in this study, equal weight is given to each base model, also in the supervised case. When new chunks of personal streaming datasets are available, phase 2 is repeated: Features are extracted from it, they are labeled and new base models are trained and added to the ensemble. Every time when a new base model is added to the ensemble, the ensemble model better adapts to the user’s personal moving style by becoming more personal, and therefore, more accurate.

## 4. Selecting the Threshold for the Semi-Supervised Approach

As Figure 1 shows, the labeling of the incoming streaming data can be done with three different approaches: non-supervised, semi-supervised, and supervised. The semi-supervised approach was originally introduced in [19]. The idea is that predicted labels with high posterior and data related to them are used in the model update process as they are, but labels with a posterior confidence below some threshold, th, are considered as uncertain, and they are labeled by the user before they are used in the model updating process. Moreover, as the studied activities are of long-term [26] nature, it can be assumed that also the windows right before and after have the same class label as the window labeled by the user. In this study, it is assumed that if the label of window *w* is *a*, then also windows w−2,w−1,w+1, and w+2 belong to class *a*. This way, one user input gives information to more than one window, improving the accuracy of the labels used to update models.

When the semi-supervised approach to personalize the recognition process is used, one key element in a successful personalization process is to define an appropriate threshold th. Too small of a threshold leads to situation where the system requires human input too often. Moreover, too large of a threshold should be avoided as well, as then the system does not rely on human intelligence as often as needed, and there is a danger of using false labels in the model updating process. In [19] two threshold values (0.95 and 0.90) for posteriors were tested. These values were used because of the experiences obtained in [27], where user-independent models for human activity were trained, and it was studied when the prediction of user-independent model is not reliable. When these threshold values were used in [19], it was noted that using these values, on average, a bit over 10% of the posteriors were below the threshold. However, when the personalization process was studied in more detail, it was noted that the initial ensemble classifier, which contains only user-independent base models, produces much less posterior values below th than an ensemble, which contains at least one personal base model.

In fact, it was noted that this share is highly unevenly distributed between predictions made using user-independent and slightly personalized models. For instance, when Shoaib data from the arm position was studied using the LDA classifier, 10.1% of the posterior values were below the threshold (using th=0.95). However, in this example during the first update round, when the ensemble contained only user-independent base models, 1.6% of the posterior were below the threshold, while during the second update round, when the ensemble contained also personal models, already 18.5% of the posteriors were below the threshold. This means that user-independent models tend to provide much higher posteriors than personalized models, as already a small number of personal data can provide so much extra information to the model that the model can better tell which predictions are reliable and which are less reliable.

**Figure 2:**
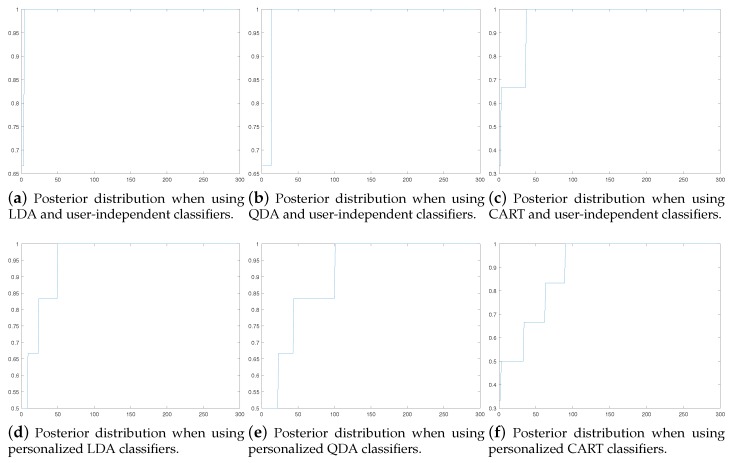
Posterior distributions obtained using user-independent and personalized models are different. In the case of personalized models there is much more variation in the posterior values. Posterior value is shown in the *y*-axis and the *x*-axis shows the number observations having posteriors smaller than the corresponding *y* value. Linear discriminant analysis (LDA), quadratic discriminant analysis (QDA), and classification and regression tree (CART).

Figure 2c,f show the posterior distribution of the user-independent model and a personalized model, which contains three user-independent base models as well as three personal base models. Based on these figures it can be noted that it is not wise to use always the same threshold value th. Instead, in the first place, when the classification is based on the user-independent model, it is wise to use a high threshold and when personal streaming data is obtained, and personal base models are added to ensemble, the used threshold should be lower. Therefore, it was decided that in this article the threshold value of th=0.95 is used when the ensemble consists only user-independent models and the threshold value of th=0.75 is used when the ensemble includes at least one personal base model.

Using this approach, for instance when Shoaib data from the arm position was studied using the LDA classifier, during the first update round, when the ensemble contained only user-independent base model, again 1.6% of the posteriors were below the threshold while during the second update round, when the ensemble contained also personal models, only 10.6% of the posteriors were below the threshold (previously 18.5%), see Table 1. Therefore, using this new method the number of required user inputs on different updating rounds is much more balanced than when always using the same threshold value.

## 5. Experimental Setup

**Figure 3:**
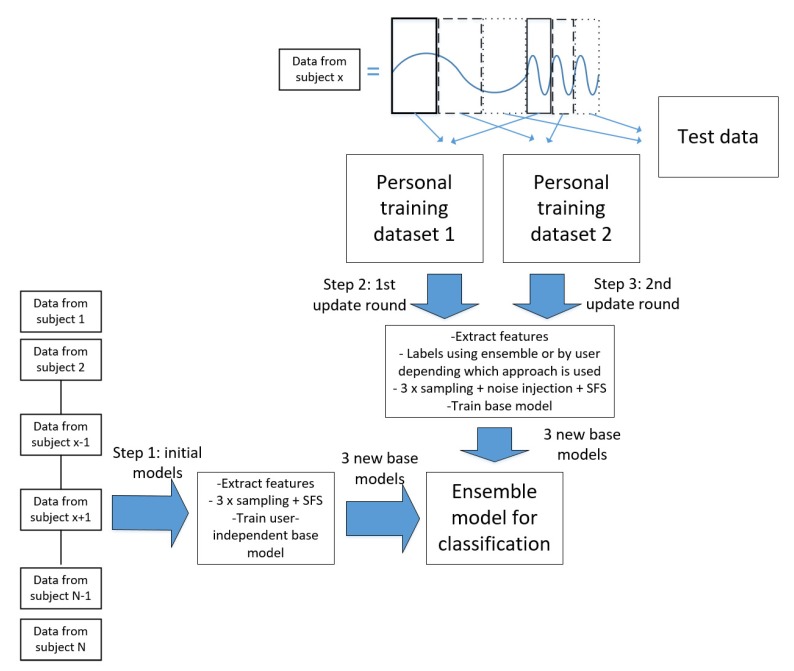
For the experiments, personal data is divided into three parts: The first two are used to update the ensemble classifier and the third part is used as test dataset.

The experimental setup used in the experiments is shown in Figure 3. The dataset contains data from *N* study subjects. In the experiments the leave-one-out method is used: Model training starts from user-independent data sets (in the Figure 3, data from subjects 1, 2, …, x−1, x+1, …, N−1, *N*, where N>x), including data from all study subjects except one. Data from one person (subject *x* in Figure 3) in turn is used for personalization and testing. Subject x’s data are divided into three totally separate parts (solid, dashed, and dotted lines) before extracting features, and to avoid over-fitting, they do not contain any overlapping. Moreover, each part contains the same amount of data from each activity, and therefore, the size of each part (solid, dashed, and dotted lines) is the same. For instance, in the example shown in Figure 3, the data of Subject x contains data from two activity classes, and both of these activity signals are divided into three separate parts. Two parts (solid and dashed) are used for personalizing the recognition model and one part (dotted) is for testing. Moreover, in should be noted that due to size of the data sets, it was decided to divide the data to three parts (two for training and one for testing). However, with bigger data sets, the data set could be divided into more than three parts.

Step 1 to train the Learn++ based recognition model is to randomly sample data from subjects 1, 2, …, x−1, x+1, …, N−1, *N* to build a user-independent base model. The best features for this are selected using SFS (sequential forward selection). The new base model is then trained based on those features and it is added to the ensemble of models. the ensemble model is then tested using the test data set, which is subject x’s data bordered with a dotted line. In these experiments, sampling with replacement is used as a sampling method, and the number of sampled instances is *S*, where $S=|S_{1}\left|+\right|S_{x-1}\left|+\ldots +\right|S_{x+1}\left|+\right|S_{N}|$ and $|S_{i}|$ is the size of the data set of subject *i*. Step 1 is repeated three times.

Step 2 is to start personalizing the ensemble model by extracting features from the first part of subject x’s data, which is bordered with a solid line. This data is labeled using the non-supervised, semi-supervised, or supervised approach. In this experiment, a problem with the data chunks used to personalize the recognition process is that they are rather small. Thus, they do not contain much variation leading easily to over-fitted base models. To avoid over-fitting, the noise injection method presented in [28], is applied to the training data sets to increase the size of training data and increase its variation. After this, a data set used in model training is selected based on random sampling (in this case, sampling with replacement and the number of sampled instances is $|S_{t_{1}}|$, where $S_{t_{1}}$ is the personal training data set 1 from subject x), and SFS is applied to it to select the best features. The new base model is then trained using the selected features and added to the ensemble model, which is again tested using subject x’s data bordered with a dotted line. Step 2 is repeated three times to provide more variability to base model set.

Step 3 is to do the same with subject x’s data, bordered with a dashed line. Also Step 3 is repeated three times. Each time when a new base model is added to the ensemble, the accuracy of the ensemble is tested with the same personal test data. In this experiment, the ensemble eventually consists of nine base models, three user-independent and six personal, so the ensemble accuracy is tested nine times.

## 6. Results and Discussion

**Figure 4:**
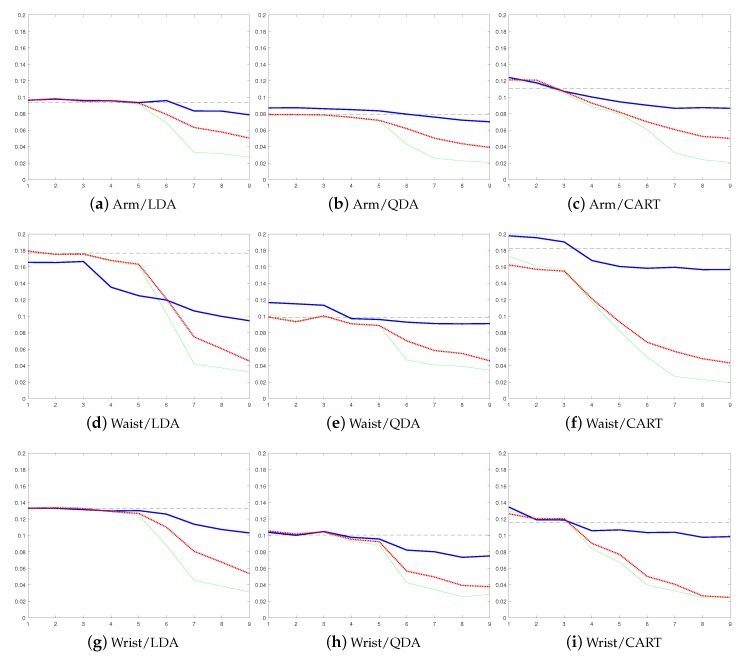
Results using Shoaib data from three body positions: arm, waist, and wrist. Adding new base models to Learn++ decreases the error rate (=1-balanced accuracy). The error rate is shown in the *y*-axis and the *x*-axis shows the number of used base models. The first row shows the results when the sensor position is the arm, the second from the waist position, and the third from the wrist position. The first column shows results using LDA, the second, QDA, and the third, CART. The results of the static user-independent model are shown using a horizontal line, the non-supervised using a blue solid line, the supervised with a green solid line, and the semi-supervised with a red dotted line.

**Figure 5:**
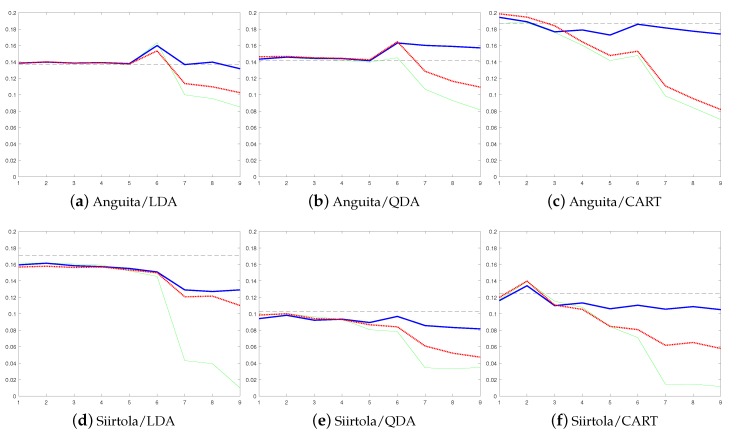
Results using the Anguita and Siirtola datasets. Adding new base models to Learn++ decreases the error rate (=1-balanced accuracy). The error rate is shown in the *y*-axis and the *x*-axis shows the number of used base models. The first row shows results using the Anguita dataset, the second using the Siirtola dataset. The first column shows results using LDA, the second, QDA, and the third, CART. The results of the static user-independent model are shown using a horizontal line, the non-supervised using a blue solid line, the supervised with a green solid line, and the semi-supervised with a red dotted line.

Non-supervised, semi-supervised (with threshold values th=0.95 when the ensemble contains only user-independent models and th=0.75 when ensemble contains at least one personal base model), and supervised approaches to personalize recognition models were tested. These are compared to the recognition rates of a static non-adaptive user-independent model. Figure 4 shows the error rates from the balanced accuracies averaged over all nine study subjects using the Shoaib dataset and how the error rate develops when base models are added to the ensemble. Balanced accuracy was used instead of accuracy as some of the used datasets are imbalanced, and therefore, accuracy is not a good performance metric in this case. Figure 5 shows the same information for Siirtola and Anguita datasets. In addition, average detection rates of different approaches, datasets, and classifiers are compared in Table 1.

**Figure 6:**
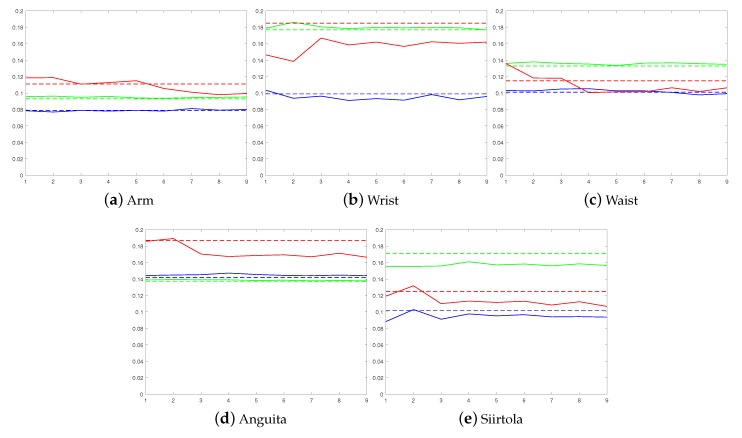
Adding new user-independent base models to the ensemble does not improve the error rate as adding personal models does. Results using the LDA classifier are shown using green, QDA classifier results are shown using blue, and CART classifier results are shown using red. Solid lines show how the error rate develops when more user-independent models are added to the ensemble and the horizontal dashed lines sho the results of the static user-independent model.

Based on these results, the benefit of using a personalized model is obvious: The average error rate starts to drop when personal models are added to the ensemble, and eventually, in each case, the average error rate using a personalized model is lower than using a user-independent model when semi-supervised and supervised personalization approaches are used, no matter which base classsifier is used. To show that this improvement is due to personal models that are added to an ensemble, as a comparison, we tested how the error rate develops if more user-independent models are added to the ensemble instead of personal models, see Figure 6. The figure shows that adding new user-independent base models to the ensemble does not have any significant effect on the error rate. Therefore, it can be concluded that the improvement obtained in Figure 4 and Figure 5 is due to personalization.

**Figure 7:**
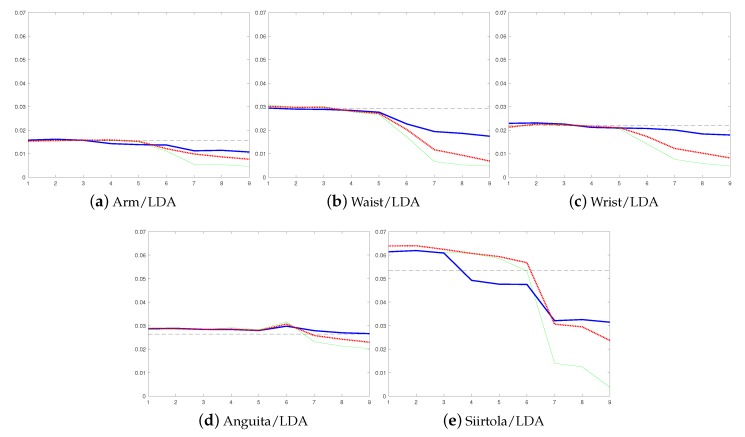
Results using the LDA classifier and false negative rate as a performance metric. Adding new base models to Learn++ decreases the false negative rate, which is shown in the *y*-axis and the *x*-axis shows the number of used base models. The results of the static user-independent model are shown using a horizontal line, the non-supervised using a blue solid line, the supervised with a green solid line, and the semi-supervised with a red dotted line.

Moreover, according to Table 1, the improvement obtained by personalization is in some cases huge, over 10% of units (see Table 1 for instance, Shoaib data from the waist using LDA and Anguita data using CART). In addition, already the non-supervised approach reduces the average error rate (87.0% vs. 89.1%). In fact, the recognition rate of the non-supervised approach is lower than the detection rate obtained using the user-independent model in only one case (Anguita dataset with QDA classifier, Figure 5b). Furthermore, to show that the improvement is not dependent on the used performance metric, results using the LDA classifier and false negative rate as a performance metric were calculated for each dataset, see Figure 7. These results are similar to the ones obtained using balanced accuracy as the performance metric. They show that personalization improves the model performance in each case compared to the user-independent model, and that the results using the supervised approach are not much better than the results obtained using the semi-supervised approach.

The results show that model update clearly benefits from human–AI collaboration as the error rates obtained using the semi-supervised approach are much lower than the ones obtained using the non-supervised approach (on average 89.1% vs. 94.0%), which was presented in our earlier work [15]. There is only one exception, where the difference between the semi- and the non-supervised model update approaches is not significant (Siirtola dataset using LDA classifier, Figure 5d). The difference between these approaches is especially large in cases where the user-independent model, originally used in the recognition process, is not that accurate, see for instance Figure 4e,f and Figure 5c. In these cases, the error rate of the semi-supervised approach starts to rapidly drop when personal base classifiers are added to the ensemble, while in the non-supervised approach, it does not drop that much. The reason is that, in these cases, a user-independent model cannot detect some of the classes at all. Therefore, the labels used to update the non-supervised model contain too many errors and the non-supervised model starts to suffer from drastic concept drift, and eventually, it cannot recognize some of the activities at all. The problem is much smaller when the semi-supervised model is used, as it can recover from similar situations thanks to user inputs, and so the number of false labels is much smaller. In fact, user-inputs can be considered as a safeguard against performance decrease caused by inaccurate labels provided by AI.

The comparison of semi-supervised and supervised approaches shows that in most of the cases the results using the semi-supervised approach are almost as good as the ones obtained using the supervised approach, which relies purely on labels provided by human intelligence (on average 94.0% vs. 96.5%). What makes this more impressive, is that when the semi-supervised approach is used, the user needs to label only about every 15th instance (and well over 90% of the labels used in the model updating process are predicted automatically when classification is based on LDA and QDA classifiers, and over 80% of the labels are predicted automatically when using the CART classifier), see Table 2. This shows that by replacing a small number of instances with correct labels, almost as high recognition rates can be achieved as by labeling all the instances. Moreover, also the results shown in Figure 7 using the false negative rate as the performance metric supports this finding.

When the Shoaib dataset is studied in more detail, it can be noted that the proposed method to personalize the human activity process is not dependent on the sensor position (see Figure 4). Moreover, it can be clearly seen from Figure 4 how the error rate gradually decreases when more personal base models are added to the ensemble. This is also the case when the results from Siirtola dataset are studied (see Figure 5) where especially semi-supervised and supervised approaches benefit from the new personal base models.

The results obtained with the Anguita dataset are not as convincing as the ones obtained using the other two datasets used in the experiments. The reason for this is that Anguita dataset is much smaller than the Siirtola and Shoaib datasets when it comes to the amount of data per subject per activity. For instance, the Anguita dataset contains only 30 s of walking data per study subject while the Siirtola and Shoaib dataset have much more walking data per study subject, for instance the Shoaib dataset has at least 3 min of walking data from each subject. In the case of Anguita data, the small amount of data means that the model does not have enough information to properly adapt to the new user’s moving style, and therefore, the error rate of the ensemble does not decrease as rapidly as with other two datasets. This is the most evident in the case of the non-supervised approach, which does not provide any advantage to the user-independent model. In fact, in the case of the QDA classifier, the error rate of the non-supervised approach is even higher than the user-independent model’s error rate, see Figure 5b. This example shows that incremental learning based on personalization requires enough data to work as it should, and fast adaptation is not as efficient as slower adaptation. However, it can be seen that small datasets also benefit from non-supervised and supervised personalization.

The main problem in our previous article [19], was that the number of needed user inputs was not evenly distributed between the update rounds. However, in this article, the threshold for the semi-supervised approach was defined a bit differently. In the previous article, the threshold for user inputs was constant while in this article the threshold value is higher when the ensemble contains only user-independent models and lower when it contains at least one personal base model. According to Table 2 this new approach improves the situation. With the CART classifier, the user needs to label 18% of the labels with both update rounds, and thus, adding personal base models to the ensemble does not increase the demand for user defined labels. With LDA and QDA, the user needs to label 6.6% and 7.7% of the labels, respectively. However, it should be noted that it was assumed that also the instances right before and after the user labeled window have the same class label as the window labeled by the user. This effectively increases the number of correct labels without disturbing the user as the total percentage of the labels replaced based on user inputs is much higher than the percentage of the windows that the user actually labeled, see Table 2, where the percentage of modified labels is presented in parentheses.

In the cases of the LDA and QDA classifiers, the need for user inputs is low, however it is still unevenly distributed between the update rounds, though not as heavily as before. Moreover, though the need for user inputs is still unevenly distributed, the need for user inputs is still much lower when using LDA (the user needs to label 2.1% of the instances during the first update round, and 11.1% during the second updating round) or QDA (4.2% during the first round and 11.3% during the second round) than when using CART (18.6% during the first round and 18.0% during the second round). However, when using CART much more user inputs are needed than with LDA and QDA, still the results with the semi-supervised approach obtained using LDA and QDA are almost as high as the ones obtained using CART (92.8% using LDA, 94.4% using QDA, and 94.8% using CART). Moreover, as CART requires more user inputs on the first update round, in many cases, using it the error rate also starts to drop more rapidly than when using LDA or QDA. This is especially visible with the Siirtola and Anguita datasets: Figure 5 shows that the error rate using LDA and QDA does not start to properly drop until the seventh base model is added to the ensemble, while when using CART, the error rate starts to drop already after fourth base model is added. In addition, while the number of required user inputs is not significantly huge, already the required number of inputs can be frustrating to the user. Therefore, it should be studied how this online labeling should be implemented to the application. One promising solution for this is presented in [29] where a dialogue-based annotation system for human activity recognition is studied. In fact, in the study it was shown that users felt more comfortable with voice-inputs than with keyboard-inputs.

## 7. Conclusions

In this article, incremental learning was used to personalize human activity recognition models using streaming data. The used incremental learning method was Learn++, which is an ensemble method that can use any classifier as a base classifier. In this study three base classifiers were compared: LDA, QDA, and CART. In the first phase, the proposed approach relies on user-independent recognition models, but when the user starts to use the application, the obtained personal streaming data is used to update and personalize the model. Three different approaches to personalize recognition models were compared: non-supervised, semi-supervised, and supervised. The non-supervised approach relies purely on predicted labels, the supervised approach uses human intelligence to label the data, and the proposed method for semi-supervised learning is a combination of these two, as it is based on human–AI collaboration: It uses artificial intelligence in most cases to label the data but in uncertain cases it relies on human intelligence.

Experiments were done using three datasets, and it was noted that personalized models are much more accurate than user-independent models. In fact, they beat the user-independent model on each dataset and with each classifier. Moreover, the error rate of models starts to rapidly decrease when models are updated using personal data showing the importance of the personal training data. On average, the recognition rate using the user-independent model is 87.0%, 89.1% using the non-supervised personalization approach, 94.0% using the semi-supervised personalization approach, and 96.5% using the supervised personalization approach. The result of the semi-supervised approach is especially impressive as it relies mostly on predicted labels and only a small number of the labels were given by the user (6.6% of the observation when using LDA as a base classifier, 7.7% when using QDA, and 18.3% using CART), and still it performed almost as well as the supervised approach where user needs to label 100% of the data.

Moreover, unlike the non-supervised approach, the semi-supervised approach does not suffer from drastic concept drift in a similar way as the non-supervised approach does, and due to relying partly on human intelligence the semi-supervised approach can also recover from it. Due to this, the recognition rate of the semi-supervised approach is almost 5%-units higher than when using the non-supervised approach. Additionally, the results obtained using the false negative rate as a performance metric support these findings. They show that personalization reduces the false negative rate and that the results obtained using supervised approach are not much better than the ones obtained using the semi-supervised approach.

Future work includes experimenting with more extensive data sets to show the true potential of the proposed method. How to avoid uncontrolled growth of the ensemble size should be studied, for instance, how suitable the method presented in [30] is for this. In addition, one limitation of the proposed method is that it does not work body-independently. This means that the position of the sensor must be the same in the training and testing sets. Therefore, it should be studied how to extend this work to make it body-position independent. In addition, one part of the future work is to make experiments using a combination of discriminative and generative models in a hybrid way, as it was done in [31].

## Figures and Tables

**Table 1:** Average performance of different personalization approaches. The performance of different approaches is measured using balanced accuracy. Standard deviation between the study subjects in parentheses.

	User-Independent	Non-Supervised	Semi-Supervised	Supervised
Arm/LDA	90.7 (6.8)	92.1 (6.0)	95.0 (4.3)	97.3 (2.0)
Waist/LDA	82.3 (9.7)	90.5 (4.8)	95.5 (4.4)	96.7 (4.8)
Wrist/LDA	86.7 (7.4)	89.7 (8.4)	94.6 (4.5)	96.9 (2.6)
Siirtola/LDA	82.9 (10.9)	87.1 (9.8)	89.0 (9.2)	99.0 (0.7)
Anguita/LDA	86.3 (11.5)	86.8 (12.6)	89.8 (10.6)	91.5 (10.0)
**Mean**	85.8 (9.4)	89.2 (8.8)	92.8 (7.1)	96.3 (5.2)
Arm/QDA	92.1 (6.2)	92.3 (4.6)	96.1 (5.8)	97.9 (2.3)
Waist/QDA	90.1 (7.8)	90.9 (6.3)	95.4 (5.1)	96.6 (3.5)
Wrist/QDA	89.9 (3.4)	92.5 (4.4)	96.2 (2.6)	97.2 (2.0)
Siirtola/QDA	89.8 (9.5)	91.8 (9.1)	95.3 (5.4)	96.5 (6.9)
Anguita/QDA	85.8 (10.0)	84.3 (11.9)	89.1 (10.2)	91.9 (8.6)
**Mean**	89.5 (7.8)	90.4 (7.8)	94.4 (6.3)	96.0 (5.4)
Arm/CART	88.9 (6.1)	91.3 (6.8)	95.0 (6.7)	97.9 (2.8)
Waist/CART	81.8 (5.0)	84.3 (4.4)	95.7 (4.6)	98.1 (1.6)
Wrist/CART	88.5 (4.0)	90.1 (5.1)	97.5 (1.8)	97.5 (2.1)
Siirtola/CART	87.5 (8.8)	89.5 (9.2)	94.2 (7.7)	98.8 (1.7)
Anguita/CART	81.3 (11.6)	82.6 (12.8)	91.8 (9.0)	93.0 (9.0)
**Mean**	85.6 (7.6)	87.6 (8.2)	94.8 (6.5)	97.1 (4.5)
**Average**	**87.0 (8.3)**	**89.1 (8.3)**	**94.0 (6.6)**	**96.5 (5.0)**

**Table 2:** The percentage of user inputs required by the semi-supervised approach with different classifiers and datasets. It was assumed that also the windows right before and after the user labeled window have the same class label as the window labeled by the user. Therefore, the total percentage of the labels replaced based on user inputs is higher than the percentage of the windows that user actually labeled. The percentage of actually replaced labels is presented in parentheses.

	Mean	Round 1	Round 2
Arm/LDA	6.1 (15.3)	1.6 (6.6)	10.6 (24.0)
Waist/LDA	11.2 (25.1)	4.3 (15.3)	18.1 (34.9)
Wrist/LDA	7.8 (18.7)	2.0 (8.1)	13.6 (29.3)
Anguita/LDA	5.5 (13.1)	1.2 (5.0)	9.8 (21.2)
Siirtola/LDA	2.4 (7.4)	1.3 (5.5)	3.6 (9.2)
**Mean**	6.6 (15.9)	2.1 (8.1)	11.1 (23.7)
Arm/QDA	7.4 (19.5)	3.7 (13.4)	11.2 (25.6)
Waist/QDA	6.9 (18.1)	3.8 (14.0)	10.0 (22.3)
Wrist/QDA	10.5 (24.9)	5.1 (17.8)	16.0 (32.0)
Anguita/QDA	7.7 (18.9)	3.2 (11.5)	12.2 (26.3)
Siirtola/QDA	6.0 (16.2)	5.1 (16.7)	7.0 (15.7)
**Mean**	7.7 (19.5)	4.2 (14.7)	11.3 (24.4)
Arm/CART	15.4 (37.6)	14.2 (38.3)	16.5 (36.8)
Waist/CART	21.0 (41.3)	21.9 (43.5)	20.2 (39.1)
Wrist/CART	19.6 (39.2)	19.6 (41.4)	19.5 (37.0)
Anguita/CART	24.8 (46.5)	22.7 (46.3)	26.9 (46.8)
Siirtola/CART	10.8 (25.1)	14.6 (33.5)	6.9 (16.8)
**Mean**	18.3 (37.9)	18.6 (40.6)	18.0 (35.3)
